# Supplementary material for: OpenMM 7: Rapid development of high performance algorithms for molecular dynamics
Source: PLoS Comput Biol. 2017 Jul 26;13(7):e1005659. doi: 10.1371/journal.pcbi.1005659 (PMC5549999; doi:10.1371/journal.pcbi.1005659)
Supplement: S1 Source Code — (ZIP) [file pcbi.1005659.s002.zip › OpenMM-7.0.1-Source/docs/api-python/generated/simtk.openmm.app.charmmcrdfiles.CharmmCrdFile.html]

CharmmCrdFile — OpenMM 7.0.1.dev-5e86c4f documentation


# CharmmCrdFile¶

*class* `simtk.openmm.app.charmmcrdfiles.``CharmmCrdFile`(*fname*)¶
:   Reads and parses a CHARMM coordinate file (.crd) into its components,
    namely the coordinates, CHARMM atom types, resid, resname, etc.

    `natom`¶
    :   *int*

        Number of atoms in the system

    `resname`¶
    :   *list*

        Names of all residues

    `positions`¶
    :   *list*

        All cartesian coordinates [x1, y1, z1, x2, ...]

    Examples

    ```
    >>> chm = CharmmCrdFile('testfiles/1tnm.crd')
    >>> print '%d atoms; %d coords' % (chm.natom, len(chm.positions))
    1414 atoms; 1414 coords
    ```

    `__init__`(*fname*)¶

    Methods

    |  |  |
    | --- | --- |
    | `__init__`(fname) |  |

    `__delattr__`¶
    :   x.\_\_delattr\_\_(‘name’) <==> del x.name

    `__format__`()¶
    :   default object formatter

    `__getattribute__`¶
    :   x.\_\_getattribute\_\_(‘name’) <==> x.name

    `__hash__`¶

    `__reduce__`()¶
    :   helper for pickle

    `__reduce_ex__`()¶
    :   helper for pickle

    `__repr__`¶

    `__setattr__`¶
    :   x.\_\_setattr\_\_(‘name’, value) <==> x.name = value

    `__sizeof__`() → int¶
    :   size of object in memory, in bytes

    `__str__`¶

High performance molecular simulation on GPUs


### Navigation

- Application Layer
  - Loaders and Setup
  - Representation and Manipulation
  - Simulation
  - Reporting Output
  - Extras
- Library Layer

©2015, Stanford University and the Authors.
|
Powered by Sphinx 1.3.1
& Alabaster 0.7.7
|
Page source
